# Spinning Gland Transcriptomics from Two Main Clades of Spiders (Order: Araneae) - Insights on Their Molecular, Anatomical and Behavioral Evolution

**DOI:** 10.1371/journal.pone.0021634

**Published:** 2011-06-29

**Authors:** Francisco Prosdocimi, Daniela Bittencourt, Felipe Rodrigues da Silva, Matias Kirst, Paulo C. Motta, Elibio L. Rech

**Affiliations:** 1 Instituto de Bioquímica Médica, UFRJ, Rio de Janeiro, Brazil; 2 Pós-graduação em Ciências Genômicas e Biotecnologia, UCB, Brasília, Brazil; 3 EMBRAPA Amazônia Ocidental, Manaus, Brazil; 4 EMBRAPA Informática Agropecuária, Campinas, São Paulo, Brazil; 5 Institute of Food and Agricultural Sciences (IFAS), University of Florida, Gainesville, Florida, United States of America; 6 Departamento de Zoologia – UnB, Brasília, Brazil; 7 EMBRAPA – CeNaRGen, Brasília, Brazil; AC Camargo Cancer Hospital, Brazil

## Abstract

Characterized by distinctive evolutionary adaptations, spiders provide a comprehensive system for evolutionary and developmental studies of anatomical organs, including silk and venom production. Here we performed cDNA sequencing using massively parallel sequencers (454 GS-FLX Titanium) to generate ∼80,000 reads from the spinning gland of *Actinopus* spp. (infraorder: Mygalomorphae) and *Gasteracantha cancriformis* (infraorder: Araneomorphae, Orbiculariae clade). *Actinopus* spp. retains primitive characteristics on web usage and presents a single undifferentiated spinning gland while the orbiculariae spiders have seven differentiated spinning glands and complex patterns of web usage. MIRA, Celera Assembler and CAP3 software were used to cluster NGS reads for each spider. CAP3 unigenes passed through a pipeline for automatic annotation, classification by biological function, and comparative transcriptomics. Genes related to spider silks were manually curated and analyzed. Although a single spidroin gene family was found in *Actinopus* spp., a vast repertoire of specialized spider silk proteins was encountered in orbiculariae. Astacin-like metalloproteases (meprin subfamily) were shown to be some of the most sampled unigenes and duplicated gene families in *G. cancriformis* since its evolutionary split from mygalomorphs. Our results confirm that the evolution of the molecular repertoire of silk proteins was accompanied by the (i) anatomical differentiation of spinning glands and (ii) behavioral complexification in the web usage. Finally, a phylogenetic tree was constructed to cluster most of the known spidroins in gene clades. This is the first large-scale, multi-organism transcriptome for spider spinning glands and a first step into a broad understanding of spider web systems biology and evolution.

## Introduction

Spidroins (or spider fibroins) are proteins from spider silks that have been studied for over 50 years. In classical studies made in the 1960s, Peakall described the glandular origins of silk fibroins, the effects of drugs on the regulation of protein synthesis in spinning glands [1], and differences in the regulation of different silk glands [2]. With the advent of biotechnology, the search for spidroins is turning into a commercial venture, and researchers have predicted the transgenic expression of silk proteins known to be half as strong as steel, and slightly less flexible than nylon [3]. Their biotechnological potential are now under extensive study and these proteins may be used in a number of industrial applications – such as the production of biomaterials, fibers and textiles, films and bioplastics, hydrogels, porous sponges, and microcapsules [4], [5]. Recently, researchers have produced specialized engineered bacteria to express these large filamentous proteins, opening the door for the biotechnology industry [6].

In this context it may be surprising to realize that no spider genomes or spinning gland transcriptomes have been extensively studied to this date in an attempt to sequence and characterize precisely the proteins involved in silk production. In order to remedy this situation, the present work presents a broad analysis of the genes expressed in the spinning gland of two spiders from different and distant evolutionary clades (Mygalomorphae and Areneomorphae). More than searching and evaluating the genetic repertoire expressed in these tissues we tried to make sense on the systems biology of spider webs, correlating the analysis of molecular data with the evolution of the spinning gland anatomy and the behavior of web usage in these organisms.

Mygalomorph spiders such as *Actinopus* spp. and tarantulas forms a monophyletic group frequently considered ancient because they retain several characteristics considered to be primitive, such as the presence of two pairs of book lungs and simple spinning structures formed by 1–3 undifferentiated globular silk glands [7], [8], [9], [10], [11]. On the other hand, orb-weaving spiders (Araneomorphae's, sub-clade Orbiculariae) of species such as *Gasteracantha cancriformis* have a much more complex spinning apparatus, consisting of seven morphologically distinct glands. Six glands: (i) the major ampullate, (ii) minor ampullate, (iii) flagelliform, (iv) tubulliform, (v) pyriform and (vi) aciniform glands are responsible for silk production and (vii) the aggregate gland secretes a sticky glue. By convention, a silk protein (spidroin) is named according to the gland that produced it (*e.g.*, the major ampullate spidroin as produced by the major ampullate gland) [12]. The molecular structure of Orbiculariae spidroins primarily consists of repeats of four amino acid motifs: polyalanine (A), glycine and alanine (GA)_n_; two glycines and a third, variable amino acid (GGX, where X represents a small subset of amino acids); and GPGX(X)_n_ (P indicates proline) [13], [14], [15], [16], [17], [18]. The presence or absence of each motif in the silk determines the mechanical properties associated to different behavioral features in a spider's life [16], [19], [20]. However, these motifs are poorly represented in mygalomorph spidroins, which are characterized by a long (163 to 183 amino acids) ensemble repeat composed of a complex mixture of serine and alanine rich sequences, including a tract of threonines [21], [22]. The higher complexity of the spinning mechanism in the Orbicularia clade is related to a more complex ecological and behavioral use of various specialized silks formed by the assembly of complex and specialized repertoire of spidroin proteins. *Actinopus* spp. utilizes its primitive web mainly to cover burrows made in the ground, used for shelter and hunting. On the other hand, *G. cancriformis* is capable of building complex flat spiral webs and uses the web for a variety of behaviors, such as (i) building the web's radial support; (ii) filling the spiral part of the web; (iii) going down from trees; (iv) wrapping insects; (v) making a sticky glue; etc.

GenBank [23] contains only 2 single and partial sequences (for 18S and 28S ribosomal RNA) for the entire *Actinopus* genus. Nineteen partial sequences have been deposited for the genus *Gasteracantha*, and most of them code for ribosomal RNAs, histones and cytochrome oxidase genes. For *G. cancriformis*, there are 15 sequenced genes, including the one encoding the major ampullate spidroin 2 (MaSp2). The entire order Araneae has fewer than 29,000 sequences deposited in GenBank (as of April 2011), including the dbEST database [24]. Until now, the broadest analysis of spider transcriptomes involved the Mygalomorphae family Theraphosidae, better known as tarantulas. In 2006, a group from the University of São Paulo (Brazil) sequenced and analyzed 7,584 transcripts from the hemocytes of the tarantula spider *Acanthoscurria gomesiana*, characterizing about 1,500 new genes in this organism [25]. Using gene ontology [26] for transcript annotation, they identified an abundance of cDNAs for hemocyanin, lectin, structural constituents of ribosomes and the cytoskeleton, as well as 73 transcripts possibly involved in the spider immune response. In 2009, 2,507 5′ ESTs from the skeletal muscle of another tarantula of the genus *Aphonopelma* were produced and analyzed [27]. As expected, a significant number of skeletal muscle-related genes were found in their analysis, which supported the existence of both actin- and myosin-linked regulation of muscle contractions in the tarantula.

Here, the cDNA repertoire obtained from *Actinopus* spp. and *G. cancrifomis* spinning glands were evaluated under a strict bioinformatics methodology. Therefore, the present work represents the most extensive characterization of spider transcriptomes to date, describing 78,913 transcriptomic sequences from the spinning glands of *Actinopus* spp. and *G. cancriformis*, thus increasing over 2.5-fold the number of spider sequences available in public databases (pictures of the spiders analyzed here and their webs can be seen at the Supplementary Information S1).

## Results

### 1. EST cleaning and sequence assembly

Sequencing reads were deposited in SRA database [28] with the accession number SRA026672. The entire set of reads was analyzed using *seqclean* script from TGICL package [29] in order to proceed with trimming and validation of ESTs. Seqclean also screens for a number of contaminants, and remove low quality and low-complexity sequences (Table 1).

**Table 1:** A summary of the cleaning procedure for the transcriptomic data from spiders.

Spider	Total number of reads	Total number of bases	Number of short reads a	Number of dust reads a	Number of low quality reads a	Total number of clean reads	Total number of clean bases
*Actinopus* spp.	34,496	8,087,280	4,727	73	117	29,579	7,490,119
*G. cancriformis*	52,399	14,339,651	2,967	43	55	49,334	13,772,393

a SeqClean data.

Because sequence assembly is known to be influenced by the informational content of sequencing reads, we have tested the assembly of high-quality sequencing reads using three well-known software for EST assembly. A careful evaluation of the results was conducted before choosing the best dataset to go further on sequence characterization and similarity measures (Table 2). Internal consistence metrics based on ICI index depicted from BLAST searches were taken on account to evaluate appropriate *read to contig* mapping (see Methods). Bigger ICI values represent better consensus built based on reads' complete sequence and higher percent identity on HSP hits. The worst ICI value was shown by Celera assembly software, while CAP3 and MIRA presented better performances on ICI tests for both datasets (Table 2). A subset of reads clustered in contigs by all the three software tested have had their ICI scores averaged and once again CAP3 have shown the best results on their assembly (please check Supplementary Information S2). In a final check on internal consistence we counted the number of reads having absolutely no BLAST hits against their associated consensus sequences. This time Celera assembler have finally performed better than the others, presenting only 22 sequences without any HSP against their consensus (summing both datasets); CAP3 has shown 124 and MIRA 160 reads without BLAST to their respective consensus sequences (Supplementary Information S2).

**Table 2:** Sequence clustering and clustering consistency data.

Spider	Assembly algorithm	Number of sequences clustered	Number of contigs	Number of unigenes a	Average ICI score	Average ECI score
*Actinopus* spp.	CAP3	15,933	4,612	**18,257**	63.63	15.11
*Actinopus* spp.	Celera	13,484	4,042	20,137	59.05	13.81
*Actinopus* spp.	MIRA	15,320	5,611	19,870	63.08	19.08
*G. cancriformis*	CAP3	29,890	6,219	**25,663**	70.25	12.07
*G. cancriformis*	Celera	24,346	5,432	30,420	65.12	12.13
*G. cancriformis*	MIRA	28,988	7,783	28,129	68.78	20.50

a Contigs and singlets.

Our sequence assembly measurements benefited from another metric for evaluating the consistency of producing separated sets of sequence clusters. The external consistency index (ECI) provides insight into whether assemblers have produced well-separated sequence clusters. Although the ECI may depend on the rate of evolution of gene families and paralogization in natural species, biased indices can indicate erroneous clustering procedures. Previous works [30], [31] have shown that values higher than 75 may signify overlapping sequence clusters that are clustered incorrectly by algorithms. All of the software used in this study computed a satisfactory average ECI distance between consensus pairs (Table 2).

Therefore, CAP3 presented the best results for internal consistency, whereas Celera produced the best values (by a slight margin) for external consistency. However, the general ECI average for cluster comparison was adequate in CAP3, and because internal consistency helps ensure better consensus quality, we chose the CAP3 clusters and consensus to analyze further. Hereafter, all references to clusters or unigenes will be referred as the CAP3-generated data.

### 2. Transcriptome coverage

Each and every sequence consensus obtained by CAP3 – as well as non-clustered original reads (singlets) – were joined in a unique dataset for each organism analyzed to form the unigene datasets. In *Actinopus sp.*, starting with 29,579 cleaned reads, 15,933 of them were assembled by CAP3 in 4,612 contigs, while 13,645 singlets were added to build the complete unigene set for this spider. Together, these 18,258 unigenes (singlets + contigs) were considered to be the best representatives of individual genes found originally in *Actinopus'* spinning glands. In *G. cancriformis* database the number of unigenes found for the spinning glands sample was 25,663 (Table 2).

The NCBI Unigene database [32] contains a “set of transcript sequences that appear to come from the same transcription locus.” We compared the number of unigenes found for organisms of phylum Arthropoda to the number obtained for the spiders we analyzed. Fifteen arthropods and a single arachnid, the black-legged tick, were found in the Unigene database (http://www.ncbi.nlm.nih.gov/unigene, accessed September 2010). The three arthropod organisms with the highest number of unigenes in the Unigene database are the black-legged tick *Ixodes scapularis* (19,405 unigenes), the mosquito *Aedes aegypti* (17,419 unigenes) and the fruit fly *Drosophila melanogaster* (17,203 unigenes; please check Supplementary Information S3). Therefore, an arachnid displayed the highest number of unigenes among organisms from the Arthropoda clade. The high number of unigenes found here for both groups of spider was in accordance with the available Unigene data and provided evidence that the Chelicerata clade presents a richer repertoire of genes than the Arthropoda clade.

Due to the small size of the sequencing reads generated from 454 experiments, a number of the unigenes generated probably came from different parts of the same gene. The number of genes expressed in the transcriptomes analyzed may have been overestimated due to this phenomenon. Nonetheless, the present study analyzed a broad set of cDNA sequences from arachnid organisms, and most likely revealed the majority of RNAs expressed in the spinning glands of *Actinopus* spp. and *G. cancriformis*.

### 3. Analysis of the most sequenced genes

**Figure 1:**
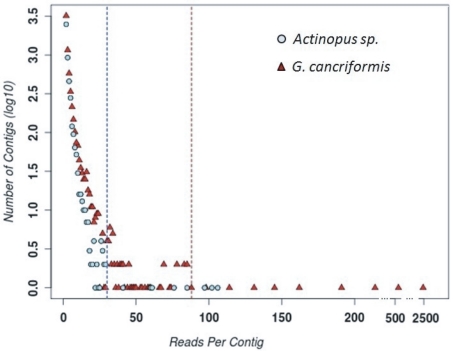
Contig sizes by number of reads assembled. The blue circles and red triangles represent data on *Actinopus spp*. and *G. cancriformis* assemblies, respectively. The vertical bars represent limit selection on the 10 most highly expressed unigenes for each spider (blue, *Actinopus spp.*; red, *G. cancriformis*).

Although normalized procedures were used to build our cDNA libraries and allowed the identification of low-expressed RNAs, the original information on the number of RNA copies expressed in spiders' spinning glands was not completely lost. Both libraries were screened for the unigenes assembled from the highest number of original sequencing reads. As expected in a well-normalized molecular library, most of our sequences were assembled into small contigs. Thus, 53.4% and 51.5% of the total number of contigs were created by assembling two single reads in *Actinopus* spp. and *G. cancriformis* datasets, respectively. Figure 1 illustrates this pattern; only few contigs contained a high number of assembled sequences. The dashed lines in Figure 1 represent a turning point on the graph, and the dots on its right represent the 10 highly sampled contigs in each species. We conducted a careful manual curation to annotate these highly sampled contigs.

Regarding the dataset for *Actinopus sp.*, 8 out the 10 highest sampled unigenes could not be identified by BLAST searches on NR database (e-value cut-off 10e-03). One of those could be clearly identified as the ribosomal protein L3, showing a very sound BLAST hit against the annotated *Ixodes scapularis* ortholog, amongst others since this protein is highly conserved. A second one matched a small part of a hypothetical protein from the scorpion *Tityus discrepans*. Others had no BLAST hits. EMBOSS package script *getorf* [33] was thus used to check for the presence of open-reading frames in those sequences. We found that 6 out these 10 putative highly expressed genes have shown to present at least one ORF bigger than 300 nucleotides (100 amino acids long), so that they probably codify proteins. In the case we accept ORFs with 50 amino acids long, all 10 genes presented them. We intend to clone and express these highly sampled genes, in order to understand their most likely function in spinning gland metabolism and web production.

A different scenario has been found for *G. cancriformis* dataset. Nine out the 10 most sequenced genes have been identified using BLAST against NCBI's non-redundant database (nr; e-value cut-off 10e-03). One gene encoded a ribosomal protein L14 and another has shown to be similar to a citochrome C oxidase-like from the hymenoptera *Glyptapanteles flavicoxis*. We also found an ATP/ADP translocase 2 similar to others from spiders and ticks. This last protein is responsible to exchange cytoplasmic ADP to ATP coming from the mithocondria. Two highly sampled genes were found to codify putative paralogs of an astacin-like metalloprotease, an ubiquitous protease found in animals with no clear function [34]. Both genes also presented about 40% identity with an astacin-like protein from the spider *Loxosceles intermedia*. These proteases have been shown to be highly expressed in *L. intermedia* venom gland [35]. The other four highly expressed unigenes identified have proven to be hypothetical conserved proteins with unknown function. They have been identified as putative targets for post-genomics analysis of gene function.

### 4. Search for similarities in protein databases

**Figure 2:**
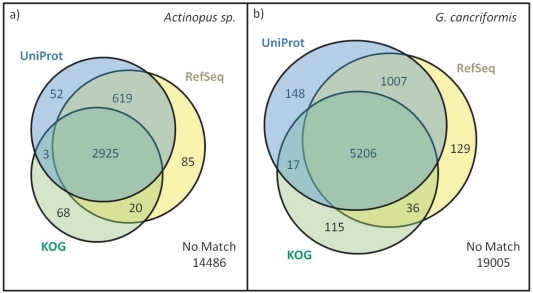
BLAST results of spider unigenes against secondary databases. a) Results for *Actinopus spp.* unigenes; b) Results for *G. cancriformis* unigenes.

To identify known genes and quantify new genes found, a BLAST analysis of the entire set of unigenes was performed against four of the most used databases (see below) in genomics and bioinformatics. Surprisingly, only 21% and 25% of the unigenes from *Actinopus* spp. and *G. cancriformis* datasets, respectively, showed any similarities with other protein-coding genes described in these databases (e-value cutoff  = 10e-05, Figure 2).

The NCBI nr, UNIPROT and RefSeq [36], [37] protein databases are continuously updated and enriched, whereas the KOG database is updated less frequently. This discrepancy explains why higher numbers of unigenes, 619 and 1,007, had no similarities in KOG for the *Actinopus* spp. and *G. cancriformis* datasets, respectively. However, more than 80% of the proteins found in other databases were also present in KOG's 2003 version [38]. RefSeq, NCBI nr, and UNIPROT all behaved similarly toward subject databases with which a similar number of unigenes was aligned. The reproducibility of results in different data resources highlights the robustness of the analysis. Figure 2 displays the sharing of unigene hits among the curated databases. BLAST hits against the non-curated nr database produced similar results, with 3,692 unigenes identified for *Actinopus* spp. and 6,498 for *G. cancriformis*.

The extremely high number of sequences for which BLAST found no matches, more than 79% and 74% of the unigenes of *Actinopus* spp. and *G. cancriformis*, respectively, can be explained by the limited sequence data available for organisms in the Araneae order. The evolutionarily closest organisms whose complete genomes are complete are insects, such as *Drosophila* [39], *Aedes* [40] and *Anopheles* spp. [41]. Those organisms belong to a different subphylum of Arthropoda from the spiders in the present work; therefore, the identification of a large number of new genes was not unexpected.

We carefully analyzed the unigenes with no BLAST hits in the secondary, curated databases in order to discover whether they were shared between the two spiders studied. A strict BLAST search was performed between the *Actinopus* spp. and *G. cancriformis* no-match unigenes (NMUs). We found 5,476 NMUs from *Actinopus* spp. with a BLAST hit (e-value cutoff  = 1e-10) against *G. cancriformis* NMUs. The reverse search returned 5,493 *G. cancriformis* NMUs with putative orthologs in the *Actinopus* transcriptome. This result indicates the presence of previously unknown hypothetical genes conserved among spider genomes.

The high number of NMUs with no match even between the spiders studied may represent species-specific or clade-specific transcripts. The EMBOSS package *getorf* was used to check for the presence of open reading frames (ORFs) in the NMUs. Getorf was conducted using two ORF size cutoffs. When we used a size cutoff of 100 nucleotides, 6,009 and 7,088 ORFs were encountered in the NMU datasets of *Actinopus* spp. and *G. cancriformis* spiders, respectively, with an average of more than one ORF per unigene. When we raised the parameter to 300 nucleotides, 161 and 335 ORFs were found in the NMU datasets of *Actinopus* spp. and *G. cancriformis*, respectively. Considering that unigenes are formed by only partial RNA sequences, we predict that the vast majority of these unmatched sequences will encode proteins or small clade-specific peptides.

### 5. Search for antisense RNAs

Sequencing reads used to build unigenes may have originated from the transcription of either sense or antisense DNA strands. It was recently discovered that antisense RNAs are involved in the fine tuning of eukaryotic transcription [42], [43]. With this in mind, reads forming consensus sequences were searched to establish the level of antisense transcription control in spiders. We found 1,658/4,544 contigs with at least one antisense transcript in the *Actinopus* spp./*G. cancriformis* datasets, which represents 36%/73% of all contigs analyzed. *G. cancriformis*, therefore, appears to frequently use this process for transcript turnover. In terms of the absolute number of antisense transcripts observed in the contigs, we found 2,998/31,530 antisense reads in the *Actinopus* spp./*G. cancriformis* tuple. These numbers provide further evidence of the broader use of antisense transcripts in Araneomorphae compared with spiders from the infraorder Mygalomorphae, suggesting that the first clade is represented by more complex organisms not only in terms of anatomical and behavioral characteristics, but also regarding molecular regulatory mechanisms. These numbers are also in accordance with the evidence found in previous works that complexification of animal behavior may account for RNA-based regulatory networks [44], [45].

Antisense transcripts were found for genes that encode spidroin sequences in *G. cancriformis*. Supplementary Information S4 shows a putative spidroin contig (the MaSp2 paralog) containing plus and minus strand reads.

We also investigated the relative proportions of sense and antisense transcripts. When the genome of the spiders under analysis is not known, it is difficult to predict the correct sense of transcripts. Therefore, transcripts were considered antisense if they differed in strand from the majority of other transcripts in the same cluster. The CAP3 assembler provided the information on the reads' strands for each cluster. Approximately one third of the contigs presented identical numbers of sense and antisense transcripts, or 636 and 1,363 contigs for *Actinopus* spp. and *G. cancriformis*, respectively.

### 6. Comparative transcriptomics of Araneae

Two other datasets containing cDNA sequences from the Mygalomorphae tarantula species *A. gomesiana* (6,790 sequences) and *Aphonopelma* spp. (Theraphosidae family) (2,697 sequences) were retrieved [25], [27]. The *A. gomesiana* and *Aphonopelma* spp. sequences were assembled using CAP3 software under the same conditions described above for *Actinopus* spp. and *G. cancriformis* reads. The sequence clustering of the *A. gomesiana* and *Aphonopelma* EST sequences resulted in 983 and 145 contigs, respectively, to create a total of 3,641 and 775 unigenes, respectively. Thus, these four spiders' unigene datasets were locally aligned against each other in a pairwise fashion. The numbers of highly similar BLASTN hits (e-value cutoff  = 1e-10) were counted and analyzed (Table 3).

**Table 3:** Pairwise BLAST analysis among Araneae unigene transcriptome datasets.

*Query*	BLAST Database (subject) a			
	*Actinopus* spp.	*G. cancriformis*	*Aphonopelma* spp.	*A. gomesiana*
*Actinopus* spp.		6,884 (7,245)	355 (213)	1,113 (767)
*G. cancriformis*	7,354 (7,001)		191 (137)	503 (378)
*Aphonopelma* spp.	210 (355)	132 (171)		257 (287)
*A. gomesiana*	751 (1,083)	356 (470)	294 (264)	

a Numbers in italics represent the number of sequences matching the database (query genes). Numbers in parentheses represent the number of genes matched on the database (subject genes). A BLASTN e-value cutoff of 1e-10 was used. All searches were performed using CAP3 unigenes for organisms' assembled ESTs and singlets.

The upper and lower diagonals in Table 3 confirm each other's results and confirm the quality of the analysis. Use of the same dataset as query and subject allowed us to check for gene duplication and paralogization events in a particular dataset. When comparing the *Actinopus* spp. and *G. cancriformis* datasets, a smaller number of unigenes in the first spider hit a slightly higher number of unigenes in the second one. This was preliminary evidence that some *G. cancriformis* genes have been duplicated, resulting in a richer repertoire of paralog genes than in *Actinopus* spp. Given that *G. cancriformis* is a member of the more derived Orbicularia clade, presents a more complex set of spinning glands and builds a complex set of different molecular-based spidroins, this result was expected and confirms the further analysis of the expansion of gene families (see Results section 8 and 9).

However, when *Actinopus* spp. unigenes were compared to *A. gomesiana* unigenes, the reverse pattern was observed. The numbers in Table 1 indicate that *Actinopus* spp. presented a higher number of paralog genes than *A. gomesiana*. The same pattern observed for *A. gomesiana* was found for the other Theraphosidae spider (*Aphonopelma*), suggesting clade-specific patterns. Some of the divergence in the expected numbers may be due to the poor sample of transcripts available for the Theraphosidae clade.

### 7. Inferred electronic annotation based on KOG categories

**Figure 3:**
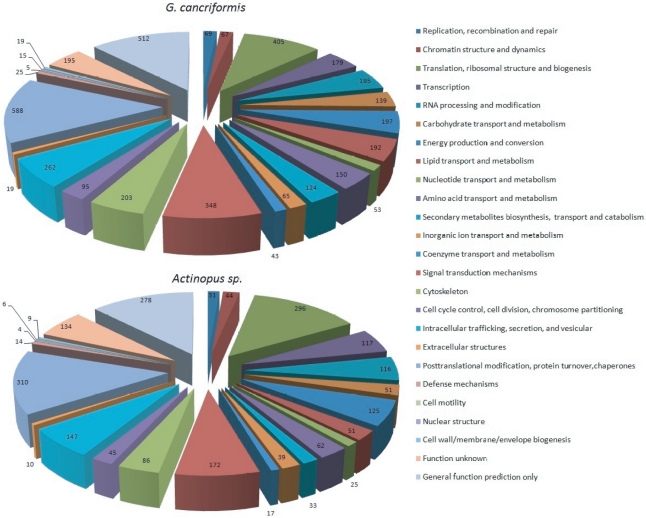
Spider genes annotated by KOG biological function. The distribution of gene classes between the two spider groups analyzed appears remarkably similar, indicating similar patterns of gene transcription in the clade.

Based on the original version for prokaryotic organisms [46], the eukaryotic version of COG presents a suitable set of ortholog groups classified into biologically meaningful categories into which organisms may be classified and annotated by sequence similarities [38]. Although Figure 2 shows a number of unigenes from the spiders analyzed that produced similarity hits in the KOG database, we have developed a stricter methodology based on the coverage of KOG gene hits and a more significant e-value to incorporate information from KOG biological classes into spider unigenes (see Methods). In this way, we were able to infer 1,533/1,952 KOG categories from 2,222/4,164 unigenes found in KOG associations for *Actinopus* spp.*/G. cancriformis*; note that some genes are multifunctional and, thus, are associated with multiple categories. Figure 3 shows the classification of genes into higher biological categories for both spiders (see Supplementary Information S5 for a complete description of unigenes matching KOG classification categories).

Figure 3 provides additional evidence for the high quality of our library regarding construction and transcriptome coverage. Genes were found for every KOG category, indicating that the metabolism of spinning glands contains the standard housekeeping elements expected for any cellular metabolism. The pie charts in Figure 3 provide evidence of a similar overall metabolism in the order Araneae. The percentages of genes found in each biological class were remarkably similar, although we were able to classify into categories almost two times more *G. cancriformis* than *Actinopus* spp. unigenes. This observation provides evidence of paralogization and gene subfunctionalization in Araneomorphae/Orbiculariae spiders. There are two gene categories found in excess in *G. cancriformis*: (i) lipid transport and metabolism and (ii) secondary metabolite biosynthesis, transport and catabolism. These data further suggest a specialization of Araneomorphae spiders in these metabolic processes, although we cannot exclude the possibility that some genes from Mygalomorphae have not been sequenced due to the randomness of library construction or that they have not been identified by BLAST searches due to their small size (the average size for *Actinopus* spp. unigenes was 282.54 bp, whereas the *G. cancriformis* average size was 316.08 bp).

### 8. Expansion of ortholog families in spider clades

One would expect that, after their separation from a common ancestor, both spiders would have molecularly and anatomically evolved inside their own lineage. We used KOG annotation to verify which spider lineage had the most genes duplicated since a common ancestor split. This test was performed using a KOG ratio of the number of genes found for a given KOG in a given dataset. A KOG ratio of 2 would mean that a given ortholog group contained two times more annotated unigenes than another group.

Only five KOGs presented a KOG ratio of at least 4 when comparing the number of paralogs in *Actinopus* spp. to *G. cancriformis* (see Supplementary Information S6). These five genes included the following: (i) the 40S ribosomal protein S29; (ii) an mRNA export protein (which contains WD40 repeats); (iii) a phosphoserine phosphatase; (iv) a nuclear distribution protein (NUDC); and (v) lectin VIP36, which is involved in the transport of glycoproteins carrying high mannose-type glycans. The expansion of these gene families in *Actinopus* spp. may have occurred in the Mygalomorphae ancestor because all but one of these described genes were also found in the *A. gomesiana* transcriptome (data not shown).

In the reverse comparison, *G. cancriformis* displayed 44 gene families with a KOG ratio larger than 4 when compared to the *Actinopus* spp. dataset (see Supplementary Information S6). Since we saw that only five KOGs were shown to duplicate faster in Mygalomorphae spiders, this result can be viewed as molecular evidence that *Actinopus* spp. retain more primitive characters than *G. cancriformis*. This molecular-based evidence confirms classical comparisons on spider groups based on anatomical and behavioral characters.

Two ortholog groups presented KOG ratios higher than 10 when comparing the numbers of paralogs in *G. cancriformis* and *Actinopus* spp., which suggested specific molecular specialization and gene duplication during the evolution of the Araneomorphae/Orbiculariae clade. Twenty-seven copies of KOG2579, which encodes “ficolin and related extracellular proteins” are annotated in *G. cancriformis*, whereas only two copies have been found in spiders of the Mygalomorphae clade (KOG ratio 13∶5). In the original KOG database [38], this ortholog group was found in 34, 10 and 6 genes in the human, fruit fly and worm genomes, respectively. Ficolins contain a signal peptide and are normally secreted. Although little is known about their function, they appear to bind carbohydrates and have opsonic activities, acting on the immune response [47], [48]. Although 27 different orthologs have been annotated as ficolin in *G. cancriformis*, these unigenes were not highly sampled. We cannot be certain what biological function this protein may have in the spinning gland or general metabolism of spiders.

Interestingly, 61 putative paralog genes for KOG3714 were found in the *G. cancriformis* dataset, while only 6 unigenes were annotated as such in *Actinopus* spp., producing a KOG ratio slightly greater than 10. This ortholog group represents proteins known as Meprin A, from a group of metalloproteases from the astacin family. Verification on the original KOG database retrieved 16, 13 and 40 genes annotated as such in the human, fruit fly and worm genomes, respectively. As shown above (see section 2.3), two of these proteins are among the ten most sampled in the *G. cancriformis* transcriptomics dataset. A new analysis of all singlets and contigs annotated as meprins has revealed 896 original reads coding for this gene, a sample representing 1.8% of all cDNAs sequenced in this spider, which suggests an important role for this gene in spinning gland metabolism. Meprins are membrane-bound endopeptidases [49], [50] and are members of a subfamily of the astacin metalloproteinases; additionally, they are distinguished from other members due to the presence of a COOH-terminal MAM domain and the absence of CUB domains [51], [52].

A number of other proteins have shown a KOG ratio larger than 4 when comparing *G. cancriformis* and *Actinopus* spp. datasets, whereas there were probably other genome duplications in an Araneomorphae ancestor. These unigenes are slightly enriched in proteins from the KOG category “O”, which indicates a higher presence of proteins involved in posttranslational modification, protein turnover, and chaperones in *G. cancriformis*. Proteins such as serine proteinase inhibitor (KU family), aspartyl protease, alpha crystallins, sulfotransferase, zuotin and related molecular chaperones, multifunctional chaperone (14-3-3 family), ubiquitin C-terminal hydrolase, asparaginyl peptidases, cysteine proteinase cathepsin L and others were probably duplicated in *G. cancriformis* since the split from their common ancestor with Mygalomorphae. A number of these proteinases and peptidases may have functions in spinning gland metabolism and may function in post-translational modifications of relevant proteins for silk production. All of these data are in accordance with the greater complexity of Orbicularia spiders anatomically, behaviorally and molecularly compared to Mygalomorphae spiders, and they provide evidence of the relevance of protein modifications on complexification of metabolism and protein evolution.

### 9. Search for silk proteins

Unigenes from both spiders were BLASTed to a manually built database of known silk proteins. The BLAST results were carefully annotated by visual inspection to certify the annotation of a given sequence as a spidroin. We paid particular attention to the repeats observed on each class of spidroins for classification (check Supplementary Information S7).

As expected, the Mygalomorphae spider had a smaller repertoire and number of spidroins than Orbiculariae, with one type of spidroin found in *Actinopus* spp. compared to six in *G. cancriformis*, reflecting the smaller complexity of its genome (Table 4). In the Orbiculariae spider, we were able to identify spidroins produced by all six glands responsible for silk production. These transcripts represented 264 reads sequenced, just over 0.5% of the almost 50,000 reads sampled in this transcriptome. The most highly represented spidroin in the library was major ampullate spidroin 2 (MaSp2), with 87 reads. According to these data, approximately 1 out of 200 proteins produced in the spinning glands is a silk protein. The corresponding value for *Actinopus* spp. was 1 of 1000, although this value is probably much higher when the glands are actively producing silk. Additionally, 114 reads for *G. cancriformis* and 45 reads for *Actinopus* spp. were classified as putative spidroins if they did not present a readable sequence for precise annotation.

**Table 4:** The classification of silk proteins found in spider transcriptomes.

Spider	Silk Protein	Number of contigs found	Number of singlets	Total number of reads
*Actinopus* spp.	Fibroin-like spidroin	2	1	5
*Actinopus* spp.	Putative spidroins	9	9	45
*G. cancriformis*	Tubulliform silk (TuSp)	2	1	25
*G. cancriformis*	Major ampullate spidroin 1 (MaSp1)	2	6	12
*G. cancriformis*	Major ampullate spidroin 2 (MaSp2)	7	11	87
*G. cancriformis*	Minor ampullate spidroin (MiSp)	4	0	10
*G. cancriformis*	Flagelliform spidroin (Flag)	2	3	8
*G. cancriformis*	Pyriform spidroin	1	0	4
*G. cancriformis*	Aciniform spidroin	1	1	4
*G. cancriformis*	Putative spidroins	21	20	114

By translating the spidroin coding sequences from contigs and singlets into proteins, we detected the presence of insertions and deletions in their nucleotide sequences. We assumed that they had been added by problems in sequencing or base-calling procedures probably caused by the repetitive molecular nature of spidroins [21]. However, it is likely that some spidroin genes will be revealed to have degenerated to pseudo-genes after genome duplications, as reported in other cases [53], [54]. In contrast, some of these duplicated genes have probably acquired new functions; a broad study of their duplication, neofunctionalization, subfunctionalization and decay associated with pleiotropy, fitness and mutational trade-offs [55] would produce an interesting molecular evolutionary story.

### 10. Molecular evolution of silk proteins

**Figure 4:**
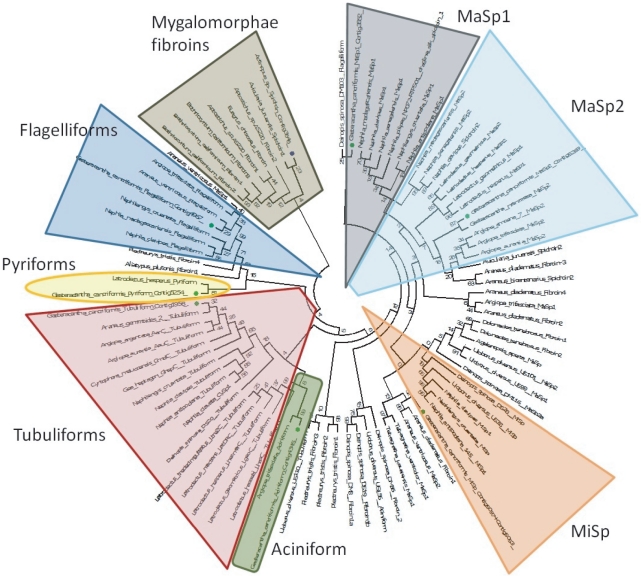
A minimum evolution tree of spider silk proteins. *G. cancriformis* sequences are marked with a green circle and *Actinopus spp*. spidroin is identified by a dark blue circle. Silk protein gene families are identified by different shapes and colors (see text for discussion).

The consensus sequence of each family of spidroins shown in Table 4 was chosen to be multiple aligned against silk proteins from a number of other spiders' species. To avoid an overestimation of deletion events in the evolution of these sequences, they were aligned using Prankster software [56], [57]. The Prankster multiple alignment of spidroin sequences was exported and converted into a minimum evolution phylogenetic analysis using MEGA software [58]. Because we have only partial sequences from a number of spidroins, the phylogeny analysis was performed using the C-terminus of the translated nucleotide sequences (Figure 4). Both *Actinopus* spp. and *G. cancriformis* annotated spidroins clustered exactly where they were expected to, evidencing that spidroins have been correctly identified with all families of known spider silk proteins found. The overall classification of spidroins shown here agrees with previous reports [22], [59], [60].

The poorer repertoire of spidroins found in the Mygalomorphae clade suggests that genome duplications that gave rise to specialized spidroins occurred after the separation between Mygalomorphae and other spider groups. However, a MaSp2-like gene has been found in the Mygalomorphae spider *Avicularia juruenses* [59]. This spidroin was formerly considered an orbicularian synapomorphy [61], and provided evidence that spidroin paralogization occurred prior to the divergence of mygalomorph and araneomorph spiders, estimated at 240 million years ago (MYA) [60]. We used the presence of MaSps in Mygalomorphae as evidence that gene duplication occurred before the anatomical specialization of glands.

Regarding the evolution of spidroin gene families, our phylogeny suggests that pyriform spidroins are the sister group of the flagelliform spidroins and that aciniforms had a more recent genetic ancestor with the tubulliform spidroins. The poor bootstrap values observed the basis of the tree may indicate that most spidroin gene families originated in an almost simultaneous event of gene duplication that probably occurred after the separation between Mygalomorphae and the other spider clades. This event resulted in further subfunctionalization and neofunctionalization of spidroin gene families in some organisms and allowed the creation and usage of different sorts of silk, some more resistant and others more flexible. For example, MaSp1 presents a number of poly-A residues that give the silk more resistance. This spidroin is mainly used to build the first radial sustentation of webs. However, the MaSp2 and MiSp spidroins contain GPG and GGS motifs that give the web its elastic and sticky properties; this type of silk is used to fill in the radial parts of the web [13], [14], [15], [16], [17], [18]. The presence or absence of amino acid motifs in spider silk may arise from different motif duplications and reorganizations in the genome, allowing this sort of molecular neofunctionalization. Once they have occurred, these rearrangements may give a specific mechanical property to the web and allow the accomplishment of different tasks in a spider's life.

## Discussion

Here, a comprehensive transcriptomic analysis was conducted for the first time to evaluate the gene expression content of spinning glands from two evolutionary distant spiders. The number of sequences evaluated in this study was more than 2.5 times larger than all the Araneae data previously deposited into the dbEST database [24] (http://www.ncbi.nlm.nih.gov/projects/dbEST/, April 2011). The sheer size of our dataset attests to the efficiency of NGS strategies for gene discovery projects, even for organisms lacking genomic information.

We were surprised that the CAP3 software [62], with its simplistic method for sequence assembly developed over 10 years ago, produced a better performance in EST clustering than more recently developed and updated software. Although the MIRA algorithm includes a complete set of parameters and metrics for working specifically with EST transcriptomic sequences produced by 454 sequencers, it was our last choice of the tested assemblers, according to evaluation metrics used. We also verified that some software works better with different clustering measures, such as the Celera assembler, which more effectively maps all the assembled reads into clusters. Most researchers would agree that the problem of sequence assembly and consensus production is highly complex and has not yet been solved, although most papers in the field simply choose a software program and proceed with assembly without further evaluations. Because sequence clustering is dependent on the informational content of the original reads, we recommend that authors test the appropriateness of the software for their own data before commencing the annotation and analysis of unigenes.

Based on the number of unigenes found here, we suspect that we have sequenced most of the coding sequences expressed by *Actinopus* spp. and *G. cancriformis* in their spinning glands. The number of unigenes in the Unigene database (http://www.ncbi.nlm.nih.gov/unigene) for the tick *I. scapularis* suggests that a number of unigenes in excess of the 25,663 found for *G. cancriformis* may be the rule for the Chelicerata clade. This excess of unigenes cannot be explained simply by the small size (318.35 bp) of the NGS-generated unigenes, although many unigenes probably represent either 3′ or the 5′ portions of genes. Our inability to identify most of the *G. cancriformis* unigenes by BLAST analysis (79% for the *Actinopus* spp. dataset) indicates a lack of interest in Chelicerata clade genomics. We tried to fix this knowledge gap in the present publication by identifying more than 30,000 new genes for this clade and making them available to the research community in the SRA database [28] (accession number SRA026672.1). The amazing genetic repertoire of spiders with their highly specialized tissues, such as the spinning and venom glands, will help researchers make biotechnological breakthroughs in the coming years, as has been recently discussed in the specialized literature [4], [5], [63].

Remarkably, we found the metalloproteases from the astacin family to be extremely significant in the *G. cancriformis* spinning gland transcriptome. Genes from this family, which still have unknown functions, corresponded to 1.8% of all transcripts sequenced in the spinning glands, a number approximately 3.6 times larger than the number of expressed spidroin genes (0.5%). Moreover, among the gene families that have undergone major amplifications since the ancestral split between the Mygalomorphae and Araneomorphae clades, we found that this family of metal-dependent proteinases has been amplified 10 times in the genome of the Araneomorphae/Orbiculariae clade. The observed proteolytic effects of metalloproteinases in the venom of some Araneomorphae spiders [35], [64], [65] are probably unrelated to their high expression in the spinning glands. Moreover, until recently, this subfamily of the meprin metalloproteases was believed to exist only in vertebrates [66], although even more recently, it was reported in the horseshoe crab *Limulus polyphemus*, an organism from the subphylum Chelicerata [67]. Our work, therefore, corroborates the evidence that this gene family is present in the Arthropoda subphylum studied here. Moreover, KOG data from 2003 have classified a number of these proteins in the genomes of invertebrates such as *D. melanogaster* and *Caenorhabditis elegans*. Meprins consist of a single, membrane-anchored member of the astacin family and have been shown to be implicated in a number of complex cellular processes in higher eukaryotes, such as cell migration, immune reactions, and tissue differentiation [68], [69], [70]. A clue to their function may come from some works reporting their relationship to human collagen fibers as they play a pivotal role in regulating interactions between cells and the extracellular matrix [71]. Researchers have recently found that these proteins are responsible for cleaving components of the extracellular matrix and suggested that they could be important players in a number of remodeling processes that involve collagen fiber deposition [72]. Do these proteins play a role in the remodeling processes of silk fiber deposition? Genetic engineering works have shown similarities between spidroin fibers and collagen fibers [73]. This hypothesis of remodeling function for meprins must be confirmed experimentally, although it is not unlikely because this process may help recycle unused web fibers. Some have reported that spiders eat their own webs to recover the energy spent on the spinning process (D. Bittencourt, personal communication), and members of *G. cancriformis* rebuild their webs daily [74].

We found a number of spider silk proteins expressed in the two transcriptomes analyzed. For *Actinopus* spp., we found only putative homologs to of the common mygalomorph spidroins. Therefore, we were not able to find clear evidence that MaSps is present in this mygalomorph spider. Although a transcriptome project is always an incomplete sample of genes, we would expect to find the expression of these proteins in the broad analysis conducted here. Recently, a spidroin that presented clear similarities to MaSp2 from the orb-weaving araneoid clade was identified in the mygalomorph spider *A. juruensis*, and phylogenetic analysis confirmed the similarities by placing this gene within the orbicularian MaSp2 clade [59]. The absence of MaSps, however, is not completely unexpected because *Actinopus* is a genus of spiders with the retention of many primitive characters, including their spinning structures [3], [10], [75]. It is conceivable that the MaSp gene family originated after the split between the Actinopodidae and Theraphosidae families of the Mygalomorphae clade. Another possible explanation for the absence of MaSps in *Actinopus* spp. is related to their ecological niches. The Amazonian *Avicularia* spp. prefer an arboreal habitat [76], and their nests are made of large leaves held together by silk in the foliage of trees. In contrast, *Actinopus* spp. is a trapdoor spider and only uses its web to build burrows or egg sacs, which require a lower mechanical strength from the silk. Interestingly, this difference also highlights the importance of gene duplication for the evolution of new biological functions.

On the other hand, we were able to verify the presence of a vast repertoire of spidroin families expressed in the complex spinning apparatus of *G. cancriformis*. Several characteristics revealed by our analysis indicate that members of *G. cancriformis* are particularly complex organisms, from the standpoint of not only anatomical characteristics but also molecular ones. For example, antisense transcripts of genes responsible for encoding spidroin sequences in *G. cancriformis* have been found, and 44 gene families presented a KOG ratio over four when compared to *Actinopus* spp. We related the number of transcripts found and the size of different glands to the overall spinning gland complex. A high number of transcripts were identified from the MaSp family, including MaSp1 and MaSp2, which are putatively expressed in the major ampullate glands, followed by the tubulliform spidroins, which are expressed in the relatively large tubulliform glands. Products of smaller glands like the flagelliform, pyriform and aciniform were also identified here. However, the expression of each spidroin must be more accurately measured before assigning the production of a specific protein to a specialized gland.

By translating the spidroin unigenes to proteins, we were able to observe the repetitive sequence of silk proteins from *G. cancriformis* (Supplementary Information S7). Although we were unable to identify the repetitive part of the *Actinopus* spp. spidroin, there is a high sequence similarity between the spidroins found in mygalomorph with some araneomorph ones. It is quite possible that the silk proteins synthesized by their common ancestor were already composed of highly homogenized tandem repeats, suggesting that this type of molecular organization is a fundamental prerequisite for silk fiber production [22], [59].

Lastly it would be interesting to evaluate the relationship between the evolution of molecular complexity and its association with the evolution of anatomical and behavioral complexity in spiders. While Actinopodidae spiders use their webs only to coat their burrows and surroundings, *G. cancriformis* and orb-weaver spiders in general use webs made from different spidroin molecules in different ecological and behavioral contexts. An intriguing question is which complexity augmenting factor arose first: (i) the anatomical; (ii) the behavioral; or (iii) the molecular? In a scenario in which behavioral modification appeared first, one can imagine that soon the spider would be under selective pressure at the molecular level to produce different types of silk with different amounts of resistance and elasticity. At that moment, the random process of gene duplication might have occurred, allowing further subfunctionalization of the spidroin genes. If this event did take place, it would probably be positively selected for. In this scenario, the last step after the increase of behavioral and molecular complexity would be the anatomical subfunctionalization, during which a new or existent part of an original spinning gland would specialize in producing a single type of silk. The finding of fibroins and MaSps in mygalomorphs [59] is an evidence that spidroin gene duplications happened before the anatomical specialization of spinning glands. Surely these hypothesized gene duplications and subfunctionalizations would need to be accompanied by differences in regulatory sequences related to the specific transcription of silk mRNAs too. This hypothesis will be better investigated after the complete sequencing of a number of spider genomes.

## Materials and Methods

**Figure 5:**
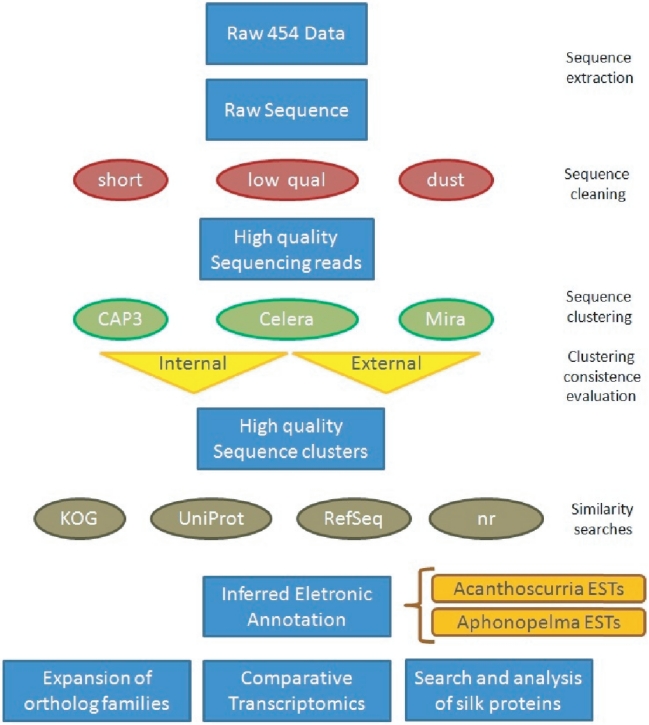
A summary of the methodology applied for transcriptome analysis of *Actinopus spp.* and *G. cancriformis* spiders.

This section describes the methods used for the analysis of the cDNA content of spider spinning glands. Figure 5 resumes the information.

### 1. Sample collection

Adult females of *Actinopus* spp. were collected along roadsides at the Cerrado border (15°55′19″ S, 47°49′45″ W; 1162 m altitude) in Brasília, DF, Brazil. Adult females of *G. cancriformis* were also collected from the Brazilian Cerrado (savanna) at the Água Limpa Farm (15°57′13″ S, 47°55′13″ W; 1130 m altitude; Brasília, DF, Brazil).

### 2. cDNA synthesis, normalization and 454 sequencing

Complementary DNA (cDNA) was synthesized and normalized as previously described [77]. Briefly, after DNase treatment of total RNA, full-length cDNAs were synthesized using the Clontech SMART cDNA Library Construction Kit (Clontech, Mountain View, CA, USA), except that the Clontech CDSIII/3′ PCR primer was replaced by the Evrogen CDS-3M adaptor (Evrogen, Moscow). The synthesized cDNA was PCR amplified for 16 cycles using the Advantage 2 Polymerase Mix (Clontech, Mountain View, CA, USA) and purified using the QIAquick PCR Purification Kit (Qiagen USA, Valencia, CA). cDNA was normalized using the Evrogen Trimmer-Direct kit (Evrogen, Moscow), following the manufacturer's instructions with modifications to remove the adapter sequences incorporated during first strand synthesis. A sequencing library was constructed from normalized cDNA (∼10 mg) and sequenced at the Interdisciplinary Center for Biotechnology Research (ICBR) at the University of Florida, following the method described elsewhere [78]. One titration run was produced using the 454 GS-FLX platform, and the nucleotide sequences were incorporated into an SFF file using 454′s software by processing the pyroluminescence intensity for each bead-containing well for nucleotide incorporation.

### 3. Reads processing

Reads were extracted from the SFF files using Blanca and Chevreux's sff_extract command line application written in python (http://bioinf.comav.upv.es/sff_extract) and stored in FASTA files. The original FASTA format sequencing reads were further cleaned using the SeqClean script from the TGICL package with a minimum size limit parameter of 70 nucleotides [29]. The SeqClean script performs automated trimming and validation of ESTs and screens for a number of contaminants, low quality and low-complexity sequences.

### 4. Clustering analysis and algorithms tested

Three strategies of sequence clustering were used: the (i) CAP3 [62]; (ii) MIRA [79]; and (iii) Celera sequence assemblers [80]. Although CAP3 is probably the most commonly used software for the assembly of Sanger reads, the MIRA and Celera programs are able to work specifically with next-generation sequencing (NGS) data. While Celera's whole-genome shotgun (WGS) assembler is popular for assembling large genomes, the MIRA software allows specific parameterization for working on EST data. In regard to parameterization, CAP3 was run in a stringent clustering mode with flags set to *-o 40* and *-s 800*, as described elsewhere [81], while MIRA was used as described in the manual for EST clustering of 454 sequences, using the flags *-job = denovo, est, normal,* and *454* [82] and Celera was used following the manual's recommendations, using flags suggested for better results for 454 sequence analysis: *overlapper = mer*, *unitigger = bog*, and utgErrorRate = 0.03 *.frg [80], [83]. CAP3 unigenes can be downloaded at http://www2.bioqmed.ufrj.br/prosdocimi/research/spider_PLoS/

### 5. Assembly evaluation

To choose the best clustered dataset following transcriptome analysis, we applied a variety of methods for internal and external consistency tests of the assemblies [30].

An internal consistency test of an assembly reports over the correct read to consensus mapping. It is tested when the original reads are aligned against their consensus sequences. In this work, the method for internal consistency was calculated using BLAST [84] to align each read to its consensus on each of the three assembly strategies tested. Furthermore, an internal consistency index (ICI) was derived by multiplying the alignment size found by BLAST by the percent identity. When reads had multiple HSPs against their contigs, a summatory function was calculated for each HSP, following the formula SUM (alignment size * percent identity). An ICI value of 100 means that the entire read was mapped with 100% sequence identity; lesser ICI values indicate a lower quality of consensus mapping. As a final internal consistency test, a subset containing only those reads assembled by all three algorithms was evaluated with regard to their ICI indexes. It should be noted that the larger the ICI score, the better the consensus generated from the original sequencing reads.

The external consistency tests evaluate whether two different contigs should be regarded as one. To perform a consistency test, the consensus sequences were aligned and the pairwise distances were calculated. The index used in this case is similar to the ICI, but while the ICI only compares the read inside the contig to its *already defined consensus sequence*, the external consistency index (ECI) of contigs is tabulated in a pairwise fashion, producing the distance between contigs. Similarly to the ICI, the ECI index is calculated by averaging in a single metric both the alignment size and the percent identity on query BLAST contigs to subject ones. Whenever reads had multiple HSPs against their contigs, a summatory was calculated for each HSP by SUM (alignment size * percent identity). A contig aligning perfectly against itself would have an ECI score of 100, whereas smaller ECI values would describe an average overlap size and identity value of sequence similarity between contigs. As with the ICI score, larger ECI values indicate sequences with greater alignment among themselves. However, when working with an ICI score, sequencing reads are ideally as close as possible to their assembled generated consensus, whereas the ECI score should not be that high. High ECI scores indicate that the consensus sequences are too close to each other; i.e., the program has separated sequences that represent the same gene and thus should be kept in the same contig. According to previous publications using a similar methodology [30], [31], we considered only contigs pairs with an ECI score higher than 75 to be problematic.

### 6. Similarity searches against primary and secondary databases

Up-to-date (as of February 2010) versions of major secondary protein databases such as UniProt and RefSeq were downloaded, as was the KOG database [36], [37], [38]. Spider unigenes were aligned using a BLASTX search against these databases with an e-value cutoff of 1e-05.

### 7. Comparative transcriptomics of the Arachnida clade

We downloaded the dbEST data for all spider species with completed transcriptome projects, which included two species in the family Theraphosidae: *Acanthoscurria gomesiana* (6,790 sequences) and *Aphonopelma* spp. (2,697 sequences). Pairwise BLAST analysis was performed using two libraries (*apho and acan*).

### 8. Inference of electronic annotation

Based on the results of the BLASTX search against secondary databases, KOG categories were incorporated into spiders' unigenes. A strict methodology was used for the propagation of information. To standardize our analysis, all other genomes compared in the present work were submitted to the same annotation pipeline. To avoid the problems that result from different groups using different methods and cutoffs for sequence annotation, we used a unique method to place all the annotations into biologically significant classes. *Bona fide* differences could thus be compared, preventing the mistakes that result from comparing non-equivalent data.

### 9. Search for silk related-genes (spidroins)

Spidroins were found via BLASTX searches against a database of silk proteins built and successfully used for this purpose in previous publications [18]. BLAST hits were manually curated to certify that the sequences were correctly designated as spidroins. All curated spidroin sequences can be downloaded from the internet at http://www2.bioqmed.ufrj.br/prosdocimi/research/spider_PLoS/.

### 10. Phylogenetic analysis of sequences

Spidroin sequences from other spiders were downloaded from GenBank. The accession numbers of sequences used for phylogenetic analysis are, per species: *Plectreurys tristis* [1 (AAK30610); 2 (AAK30611); 3 (AAK30612); 4 (AAK30613)], *Deinopsis spinosa* [1b (ABD61592); 1a (ABD61591); 2a (ABD61593); 2b (ABD61594); 3 (AAY28934.1); 4 (ABD61590), 6 (ABD61589)], *Dolomedes tenebrosus* [1 (AAK30598); 2 (AAK30599)], *Argiope aurantia* [2 (AAK30592); 3 (AAX45292)], *Argiope trifasciata* [1 (AAK30595); 2 (AAK30596); 4 (AAK30593); 5 (AAR83925)], *Argiope amoena* [2 (AAR13813)], *Argiope argentata* [3 (AAY28932)], *Latrodectus hasselti* [3 (AAY28941)], *Latrodectus hesperus*[1 (AAY28935); 2 (ABD66603); 3 (AAY28931)], *Latrodectus geometricus* [1 (AAK30602), 2 (AAK30604), 3 (AAY28940)], *Latrodectus mactans* [3 (AAY28938)], *Latrodectus tredecimguttatus* [3 (AY953078)], *Cyrtophora moluccensis* [3 (AAY28944)], *Uloborus diversus* [1 (ABD61596), 2 (ABD61599), 3 (AAY28933), 5 (ABD61598), 6 (ABD61597)], *Araneus diadematus* [1 (AAC47008), 2 (AAC47009), 3 (AAC47010), 4 (AAC47011)], *Araneus ventricosus* [1 (AAN85280), 2 (AAN85281), 4 (ABK00016)], *Araneus bicentenarius* [2 (AAC04503)], *Araneus gemmoides* [3 (AAX45294)], *Gea heptagon* [3 (AAY28943)], *Gasteracantha mammosa* [2 (AAK30601)], *Agelenopsis aperta* [1 (AAT08436)], *Nephila clavipes* [1 (AAT75312), 2 (AAT75315), 3 (AAX45295), 4 (AAF36089), 6 (AAC14589)], *Nephila clavata* [3 (BAE54450)], *Nephila antipodiana* [1 (ABC72644), 3 (AAY90151), 6 (ABC72645)], *Nephila madagascarensis* [1 (AAK30606), 2 (AAK30607), 4 (AAF36092)], *Nephila pilipes* [1 (AAV48946)], *Nephila senegalensis* [1 (AAK30608), 2 (AAK30609)], *Nephilengys cruentata* [1 (EF638446), 3 (EF638445), 4 (EF638444), 6 (EF638447)], *Tetragnatha versicolor* [1 (AAK30615)], *Tetragnatha kauaiensis* [1 (AAK30614)], *Euagrus chisoseus* [1 (AAK30600)], *Avicularia juruensis* [1 (EU652181), 2 (EU652184)], *Aliatypus plutonis* [(ABW80562)], *Aptostichus* spp. [1 (ABW80562), 2 (ABW80564)], *Bothriocyrtum californicum* [1 (ABW80565), 2 (ABW80566), 3 (ABW80567)], *Latrodectus hesperus* [FJ973621].

The evolutionary history of spider silk proteins was inferred using the minimum-evolution (ME) method [85]. The bootstrap consensus tree inferred from 1000 replicates [86] was taken to represent the evolutionary history of the analyzed taxa. Evolutionary distances were computed using the Poisson correction method [87] and are represented in the units of the number of amino acid substitutions per site. The among-site rate variation was modeled with a gamma distribution (shape parameter  = 1). The ME tree was searched using the Close-Neighbor-Interchange (CNI) algorithm [88] at a search level of 0. The Neighbor-joining algorithm [89] was used to generate the initial tree. The analysis involved 87 amino acid sequences. All ambiguous positions were removed for each sequence pair. There were a total of 199 positions in the final dataset. Evolutionary analyses were conducted using MEGA 5 software [58].

## Supporting Information

Supporting Information S1 Spiders' pictures and additional information.
(DOC)

Supporting Information S2 Sequence clustering consistence checks.
(DOC)

Supporting Information S3 Total number of unigenes per arthropod organism.
(DOC)

Supporting Information S4 Search for antisense RNAs.
(DOC)

Supporting Information S5 Inferred electronic annotation based on KOG categories.
(DOC)

Supporting Information S6 Duplicated KOGs.
(DOC)

Supporting Information S7 Repetitive sequences from *G. cancriformis* spidroins.
(DOC)
