# Supplementary material for: Spinning Gland Transcriptomics from Two Main Clades of Spiders (Order: Araneae) - Insights on Their Molecular, Anatomical and Behavioral Evolution
Source: PLoS One. 2011 Jun 29;6(6):e21634. doi: 10.1371/journal.pone.0021634 (PMC3126850; doi:10.1371/journal.pone.0021634)
Supplement: Supporting Information S1 — Spiders' pictures and additional information. (DOC) [file pone.0021634.s001.doc]

SUPPLEMENTARY INFORMATION **S1**

Prosdocimi *et al*., 2011. Spinning gland transcriptomics from two main clades of spiders (order: Araneae) - insights on their molecular, anatomical and behavioral evolution.

**Spiders’ pictures and additional information**

*Actinopus* (Figure S1.1) is a genus of spiders with an apomorphic arched head region native to South America from the infraorder Mygalomorphae, the Rastelloidina clade, and the Actinopodidae family [1]. These spiders have a rastellum, which is a reinforced part of the chelicerae adapted for digging used to build tubular burrows containing a trapdoor in the ground (Figure S1.2). Then, they mix a primitive web, soil, and plants to fabricate a protective structure [2], a revetment that isolates the burrow from adjacent substrates. *Actinopus* spp. have primitive, undifferentiated spinning glands and use their silk only to build these retreats. These burrows are used by the spider for protection from desiccation and predation during the day. At night, the spiders lie in wait behind the trapdoor, which is frequently open, and leap at preys that pass close to the burrow [2].


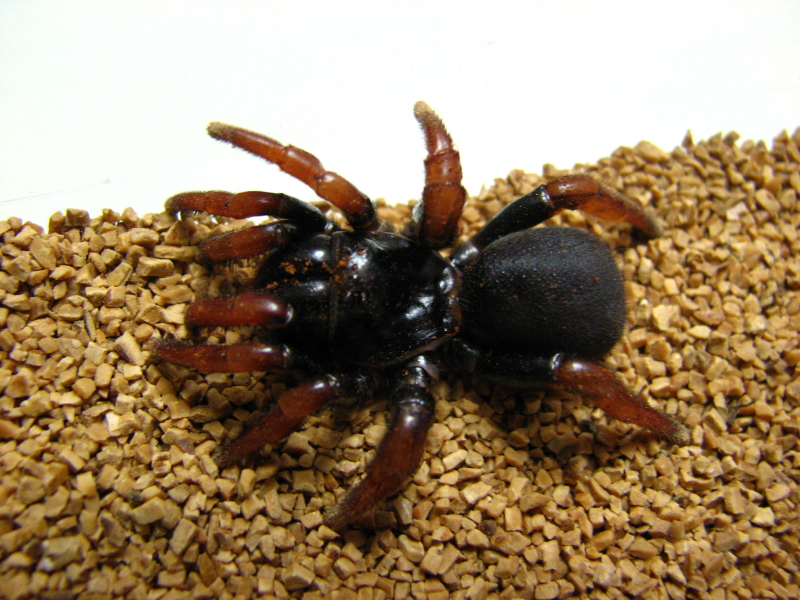


**Figure S1.1**: Picture of the spider *Actinopus spp*. (credits: EL Rech)

**Figure S1.2**: A tubular burrow made with a primitive web with a trapdoor built by *Actinopus spp.* for shelter and predation. (credits: EL Rech)

*Gasteracantha* spiders from the infraorder Araneomorphae, Orbiculariae clade, and family Araneidae build complex, flat spiral webs that are reconstructed daily and use a sticky silk for catching prey (Figure S1.3; [3]). Their spinning glands produce different types of silk made of different proteins, which are used for a number of different behaviors, such as climbing down from a tree, building the main axes of spiral webs, wrapping insects, etc. Different sorts of silk are made of different protein polymers (assembles of spidroin proteins) that give to the silk the appropriate strength and elasticity for a given behavioral usage. *Gasteracantha* spiders are commonly called spiny orb-weavers due to the presence of prominent spines on their abdomens. The upper side of these spiders’ abdomens may display colors ranging from white to red or yellow [4], with yellow being the most common. These spiders can be camouflaged as plant seeds or thorns hanging from their webs. The second species studied in this work, *Gasteracantha cancriformis*, is found exclusively in the New World. Their webs are built in large, open areas above the herbaceous layer and contain decorative silk tufts called stabilimenta.

Adult females of *Actinopus* spp. and *G. cancriformis* were collected from the Brazilian Cerrado at Brasília, DF, Brazil.


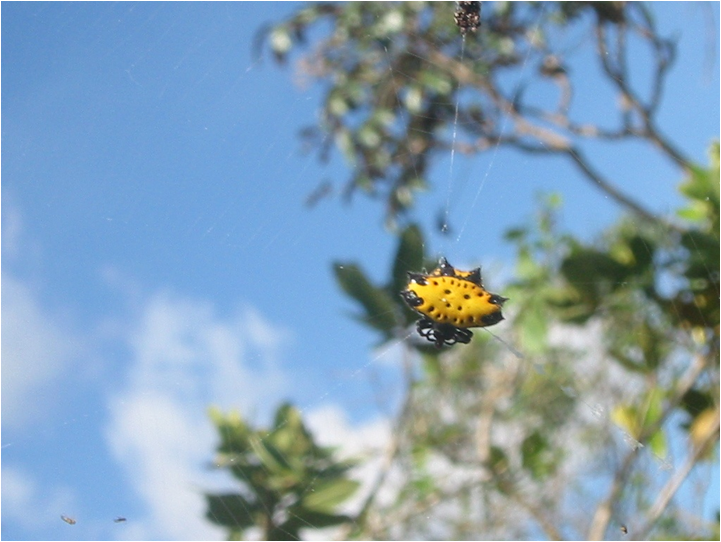


**Figure S1.3**: *Gasteracantha cancriformis* in the middle of its flat-spiral web. (credits: EL Rech)

**References***

1. Foelix RF (1996) Biology of Spiders: Oxford University Press.

2. Coyle FA (1986) The role of silk in prey capture by non-araneomorph spiders. In: Shear WA, editor. Spiders: webs, behavior and evolution. Stanford: Stanford University Press. pp. 269-305.

3. Muma MH (1971) Biological and behavioral notes on Gasteracantha cancriformis (Arachnida: Araneidae). Florida Entomol 54: 345-351.

4. Levi HW (1978) The american orb-weaver genera Colphepeira, Micrathena and Gasteracantha North of Mexico (Araneae, Araneidae). Bull Mus Comp Zoo 148: 417-442.

* Papers cited in this document only. Please check further references in the main text.
