# Supplementary material for: Spinning Gland Transcriptomics from Two Main Clades of Spiders (Order: Araneae) - Insights on Their Molecular, Anatomical and Behavioral Evolution
Source: PLoS One. 2011 Jun 29;6(6):e21634. doi: 10.1371/journal.pone.0021634 (PMC3126850; doi:10.1371/journal.pone.0021634)
Supplement: Supporting Information S2 — Sequence clustering consistence checks. (DOC) [file pone.0021634.s002.doc]

SUPPLEMENTARY INFORMATION **S2**

Prosdocimi *et al*., 2011. Spinning gland transcriptomics from two main clades of spiders (order: Araneae) - insights on their molecular, anatomical and behavioral evolution.

**Sequence clustering consistence check**

An ICI index ranging from 0 to 100 was created to verify and average the precision of reads in contig mapping (see Methods). The ICI index was averaged between the software and datasets to verify the precision of the reads in contig mapping. Table S2.1 presents the values of ICI indices for reads clustered in contigs by all software. CAP3 showed the best results of mapping reads assembled to consensus sequences.

In a final check of internal consistence, the number of reads having no BLAST hits against their consensus was counted. The Celera assembler performed better in this respect; the values are shown in Table S2.1 (last column).

**Table S2.1**: Sequence clustering information and clustering consistence evaluation data for the reads clustered in contigs by all software.

| **SPIDER** | **Clustering Algorithm** | **Number of sequences clustered by all software** | **Number of contigs^a^** | **ICI index^b^** | **Reads with no BLAST against contigs** |
| --- | --- | --- | --- | --- | --- |
| *Actinopus sp.* | CAP3 | 9633 | 3124 | 67.22 | 72 |
| *Actinopus sp.* | Celera | 9633 | 3111 | 62.44 | 15 |
| *Actinopus sp.* | Mira | 9633 | 3570 | 65.79 | 79 |
| *G. cancriformis* | CAP3 | 20274 | 4467 | 77.42 | 52 |
| *G. cancriformis* | Celera | 20274 | 4545 | 67.51 | 7 |
| *G. cancriformis* | Mira | 20274 | 5514 | 70.52 | 81 |

^a^ Number of contigs produced by the assembly these reads assembled by all contigs
^b^ Average ICI index produced from the reads assembled by all and their respective contigs
