# Supplementary material for: Spinning Gland Transcriptomics from Two Main Clades of Spiders (Order: Araneae) - Insights on Their Molecular, Anatomical and Behavioral Evolution
Source: PLoS One. 2011 Jun 29;6(6):e21634. doi: 10.1371/journal.pone.0021634 (PMC3126850; doi:10.1371/journal.pone.0021634)
Supplement: Supporting Information S3 — Total number of unigenes per arthropod organism. (DOC) [file pone.0021634.s003.doc]

SUPPLEMENTARY INFORMATION **S3**

Prosdocimi *et al*., 2011. Spinning gland transcriptomics from two main clades of spiders (order: Araneae) - insights on their molecular, anatomical and behavioral evolution.

**Total number of unigenes per arthropod organism**

The Unigene database contains the most likely number of genes found in a given organism, especially for organisms lacking genome information. Our discussion about the completeness of the two spider transcriptomes analyzed here was based on the number of unigenes found for the following arthropod organisms in the Unigene database (Table S3.1).

**Table S3.1: Unigene information for Arthropod organisms.**

| **Organism** | **Common Name** | **Clade** | **Number of unigenes** |
| --- | --- | --- | --- |
| *Ixodes scapularis* | black-legged tick | Arachnida | 19,405 |
| *Aedes aegypti* | yellow fever mosquito | Insecta | 17,419 |
| *Drosophila melanogaster* | fruit fly | Insecta | 17,203 |
| *Nasonia vitripennis* | jewel wasp | Insecta | 15,445 |
| *Daphnia pulex* | common water flea | Branchiopoda | 14,177 |
| *Acyrthosiphon pisum* | pea aphid | Insecta | 13,241 |
| *Anopheles gambiae* | African malaria mosquito | Insecta | 13,057 |
| *Bombyx mori* | domestic silkworm | Insecta | 12,458 |
| *Apis mellifera* | honey bee | Insecta | 9,749 |
| *Litopenaeus vannamei* | Pacific white shrimp | Malacostraca | 8,164 |
| *Drosophila simulans* |  | Insecta | 8,084 |
| *Glossina morsitans* |  | Insecta | 7,528 |
| *Tribolium castaneum* | red flour beetle | Insecta | 6,795 |
| *Culex quinquefasciatus* | house mosquito | Insecta | 4,957 |
| *Bicyclus anynana* | squinting bush brown | Insecta | 4,059 |

Obtained from (http://www.ncbi.nlm.nih.gov/unigene, accessed September 2010)
