# Supplementary material for: Spinning Gland Transcriptomics from Two Main Clades of Spiders (Order: Araneae) - Insights on Their Molecular, Anatomical and Behavioral Evolution
Source: PLoS One. 2011 Jun 29;6(6):e21634. doi: 10.1371/journal.pone.0021634 (PMC3126850; doi:10.1371/journal.pone.0021634)
Supplement: Supporting Information S4 — Search for antisense RNAs. (DOC) [file pone.0021634.s004.doc]

SUPPLEMENTARY INFORMATION **S4**

Prosdocimi *et al*., 2011. Spinning gland transcriptomics from two main clades of spiders (order: Araneae) - insights on their molecular, anatomical and behavioral evolution.

**Search for antisense RNAs**

A number of contigs derived from the assembly of original reads represent sequences putatively transcribed from both plus and minus DNA strands. Here, we present the alignment of some reads responsible for building Contig5814, a putative paralog gene for a spidroin protein (MaSp2) in *G. cancriformis*. Figure S4.1 shows an alignment of the reads used to build this contig. There are two antisense reads mainly encoding the 3’ portion of the sequence (F2MILYB02BYME6- and F2MILYB02B7L7J-). The presence of antisense reads probably accounts for the fine tuning of transcription/translation regulation and RNA turnover in spider proteins.


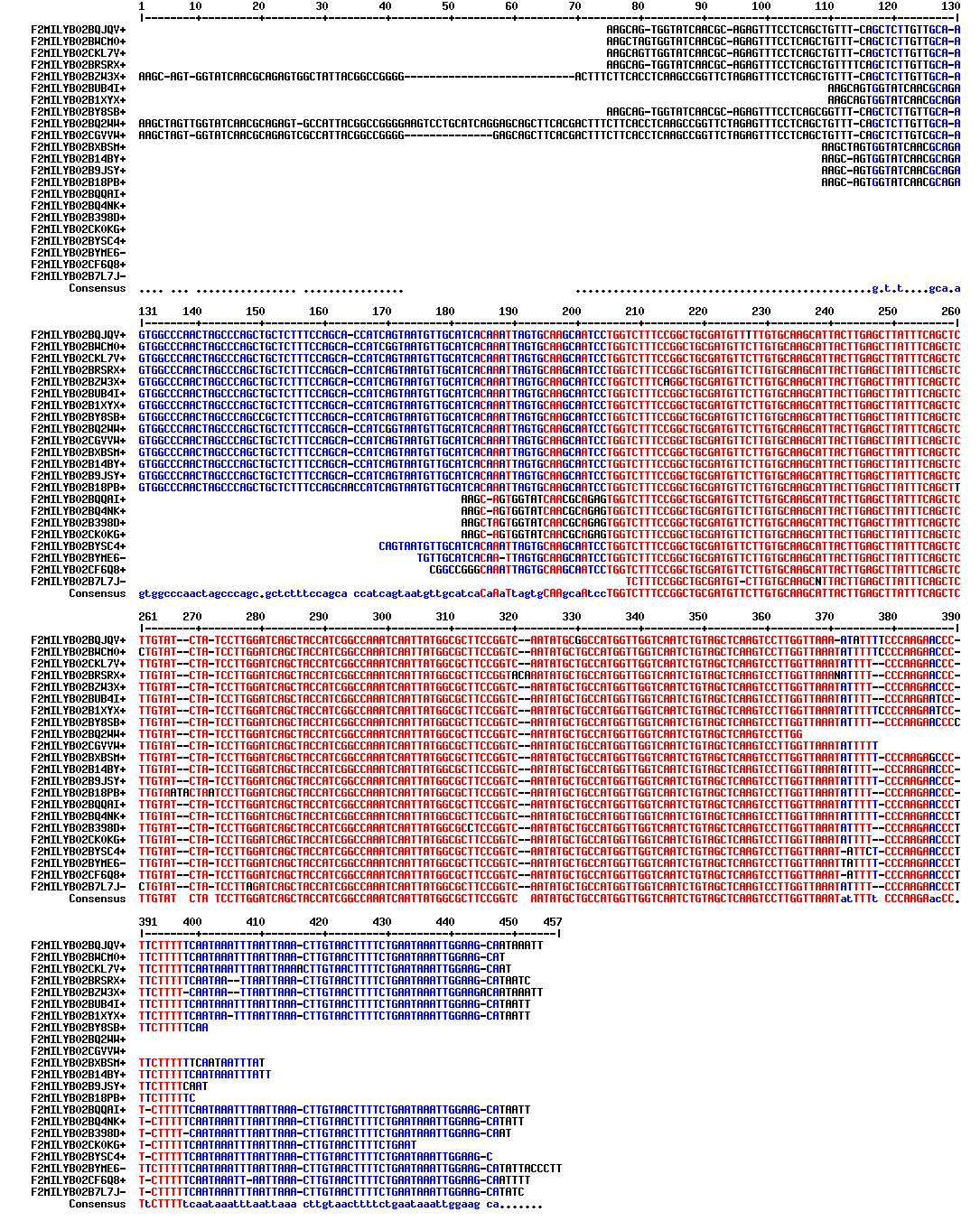


**Figure S4.1**: The alignment of several original reads used by CAP3 to build Contig5814 in *G. cancriformis*. BLAST analysis followed by manual curation indicated that this gene was a putative paralog for a silk protein gene (MaSp2).
