# Supplementary material for: Spinning Gland Transcriptomics from Two Main Clades of Spiders (Order: Araneae) - Insights on Their Molecular, Anatomical and Behavioral Evolution
Source: PLoS One. 2011 Jun 29;6(6):e21634. doi: 10.1371/journal.pone.0021634 (PMC3126850; doi:10.1371/journal.pone.0021634)
Supplement: Supporting Information S5 — Inferred electronic annotation based on KOG categories. (DOC) [file pone.0021634.s005.doc]

SUPPLEMENTARY INFORMATION **S5**

Prosdocimi *et al*., 2011. Spinning gland transcriptomics from two main clades of spiders (order: Araneae) - insights on their molecular, anatomical and behavioral evolution.

**Inferred Electronic Annotation based on KOG categories**

Unigenes of the two spiders analyzed in this work were automatically annotated into KOG categories based on stringent criteria, as were the two datasets for tarantulas obtained from dbEST (see Methods). Table S5.1 presents the information and annotations for KOG biological categories. As discussed in the main text, genes from the spiders sequenced here were found in all the KOG categories, indicating efficient transcriptome coverage. The data on tarantulas were too incomplete to allow further discussion.

**TableS5.1. Annotation of spider unigenes into KOG categories.**

| **CAT** | **KOG Category Description** | **Act Categories***^a^* | **Gas categories***^a^* | **Apho categories***^a^* | **Acan categories***^a^* | **KOG**  **SUPERCLASS***^b^* |
| --- | --- | --- | --- | --- | --- | --- |
| *L* | Replication, recombination and repair | 31 (18) | 69 (42) | 1 (1) | 28 (22) | InfoSP |
| *B* | Chromatin structure and dynamics | 44 (25) | 67 (29) | 3 (3) | 35 (21) | InfoSP |
| *J* | Translation, ribosomal structure and biogenesis | 296 (184) | 405 (188) | 26 (21) | 173 (131) | InfoSP |
| *K* | Transcription | 117 (72) | 179 (104) | 8 (7) | 86 (62) | InfoSP |
| *A* | RNA processing and modification | 116 (77) | 195 (129) | 11 (10) | 113 (69) | InfoSP |
| *G* | Carbohydrate transport and metabolism | 51 (41) | 139 (69) | 14 (12) | 59 (42) | Met |
| *C* | Energy production and conversion | 125 (81) | 197 (112) | 29 (17) | 93 (72) | Met |
| *I* | Lipid transport and metabolism | 51 (31) | 192 (70) | 3 (3) | 50 (34) | Met |
| *F* | Nucleotide transport and metabolism | 25 (18) | 53 (36) | 1 (1) | 21 (17) | Met |
| *E* | Amino acid transport and metabolism | 62 (41) | 150 (63) | 6 (5) | 66 (31) | Met |
| *Q* | Secondary metabolites biosynthesis, transport and catabolism | 33 (14) | 124 (21) | 2 (2) | 24 (14) | Met |
| *P* | Inorganic ion transport and metabolism | 39 (24) | 65 (33) | 8 (5) | 35 (23) | Met |
| *H* | Coenzyme transport and metabolism | 17 (10) | 43 (22) | 1 (1) | 19 (11) | Met |
| *T* | Signal transduction mechanisms | 172 (101) | 348 (171) | 23 (13) | 185 (89) | CelProc |
| *Z* | Cytoskeleton | 86 (40) | 203 (53) | 101 (18) | 80 (36) | CelProc |
| *D* | Cell cycle control, cell division, chromosome partitioning | 45 (27) | 95 (50) | 2 (2) | 43 (22) | CelProc |
| *U* | Intracellular trafficking, secretion | 147 (81) | 262 (142) | 15 (14) | 121 (74) | CelProc |
| *W* | Extracellular structures | 10 (6) | 19 (12) | 2 (1) | 20 (6) | CelProc |
| *O* | Posttranslational modification, protein turnover,chaperones | 310 (175) | 588 (202) | 19 (18) | 242 (141) | CelProc |
| *V* | Defense mechanisms | 14 (7) | 25 (8) | 3 (2) | 20 (6) | CelProc |
| *N* | Cell motility | 4 (2) | 5 (3) | 0 (0) | 5 (3) | CelProc |
| *Y* | Nuclear structure | 6 (5) | 15 (11) | 0 (0) | 8 (7) | CelProc |
| *M* | Cell wall/membrane/envelope biogenesis | 9 (6) | 19 (11) | 1 (1) | 10 (8) | CelProc |
| *S* | Function unknown | 134 (110) | 195 (157) | 5 (5) | 111 (100) | Poor |
| *R* | General function prediction only | 278 (159) | 512 (214) | 23 (16) | 256 (130) | Poor |
|  | **TOTAL** | **2222 (1533)** | **4164 (1952)** | **307 (178)** | **1903 (1171)** |  |

^a^ Number of KOGs found per category is shown as well as non-redundant number of different KOGs found per category (in parenthesis) ^b^ Superclasses KOG description: InfoSP (Information Storage and Processing), Met (Metabolism), CelProc (Cellular Processes and Signalling), Poor (Poorly Characterized)
