# Supplementary material for: Spinning Gland Transcriptomics from Two Main Clades of Spiders (Order: Araneae) - Insights on Their Molecular, Anatomical and Behavioral Evolution
Source: PLoS One. 2011 Jun 29;6(6):e21634. doi: 10.1371/journal.pone.0021634 (PMC3126850; doi:10.1371/journal.pone.0021634)
Supplement: Supporting Information S6 — Duplicated KOGs. (DOC) [file pone.0021634.s006.doc]

SUPPLEMENTARY INFORMATION **S6**

Prosdocimi *et al*., 2011. Spinning gland transcriptomics from two main clades of spiders (order: Araneae) - insights on their molecular, anatomical and behavioral evolution.

**Duplicated KOGs**

The annotation of spider genes into ortholog groups (KOGs) allowed us to verify which of these groups were duplicated along the evolution of spiders when comparing the *Actinopus* spp*.* and *G. cancriformis* data. A KOG ratio was developed to indicate the ratio of gene expansion per orthologous group. Table S6.1 presents the data for which the KOG ratio was larger than 4. The presence of a larger number of gene families with a higher KOG ratio in *G. cancriformis* than in *Actinopus* spp*.* is an evidence of the well-known retention of primitive characters from ancestral spiders in Mygalomorphae group. The evolution by gene duplication followed by sub/neofunctionalization of genes in *G. cancriformis* was evidenced by the data in Table S6.1 and manual analysis of silk proteins (see Results section 8 and 9 in the main text).

**TableS6.1.** KOG ratio evidence for genome duplication

| **KOG** | **Number of paralogs (Actinopus)** | **Number of paralogs**  **(Gasteracantha)** | **KOG ratio***^a^* | **KOG category** | **KOG annotation** |
| --- | --- | --- | --- | --- | --- |
| KOG0647 | 4 | 1 | 4 | A | mRNA export protein (contains WD40 repeats) |
| KOG1615 | 4 | 1 | 4 | E | Phosphoserine phosphatase |
| KOG2265 | 4 | 1 | 4 | T | Nuclear distribution protein NUDC |
| KOG3506 | 4 | 1 | 4 | J | 40S ribosomal protein S29 |
| KOG3839 | 4 | 1 | 4 | U | Lectin VIP36, involved in the transport of glycoproteins carrying high mannose-type glycans |
| KOG0002 | 1 | 4 | 4 | J | 60s ribosomal protein L39 |
| KOG0141 | 1 | 4 | 4 | EI | Isovaleryl-CoA dehydrogenase |
| KOG0796 | 1 | 4 | 4 | A | Spliceosome subunit |
| KOG0841 | 1 | 4 | 4 | O | Multifunctional chaperone (14-3-3 family) |
| KOG1205 | 2 | 8 | 4 | Q | Predicted dehydrogenase |
| KOG1348 | 1 | 4 | 4 | O | Asparaginyl peptidases |
| KOG1392 | 1 | 4 | 4 | I | Acetyl-CoA acetyltransferase |
| KOG1411 | 1 | 4 | 4 | E | Aspartate aminotransferase/Glutamic oxaloacetic transaminase AAT1/GOT2 |
| KOG1490 | 1 | 4 | 4 | R | GTP-binding protein CRFG/NOG1 (ODN superfamily) |
| KOG1543 | 6 | 24 | 4 | O | Cysteine proteinase Cathepsin L |
| KOG1762 | 1 | 4 | 4 | J | 60s acidic ribosomal protein P1 |
| KOG2046 | 1 | 4 | 4 | Z | Calponin |
| KOG2177 | 1 | 4 | 4 | O | Predicted E3 ubiquitin ligase |
| KOG2201 | 1 | 4 | 4 | H | Pantothenate kinase PanK and related proteins |
| KOG2276 | 1 | 4 | 4 | E | Metalloexopeptidases |
| KOG2309 | 1 | 4 | 4 | J | 60s ribosomal protein L2/L8 |
| KOG2408 | 2 | 8 | 4 | R | Peroxidase/oxygenase |
| KOG2597 | 1 | 4 | 4 | R | Predicted aminopeptidase of the M17 family |
| KOG2778 | 1 | 4 | 4 | O | Ubiquitin C-terminal hydrolase |
| KOG3255 | 1 | 4 | 4 | J | 60S ribosomal protein L9 |
| KOG3312 | 1 | 4 | 4 | S | Predicted membrane protein |
| KOG3656 | 1 | 4 | 4 | R | FOG: 7 transmembrane receptor |
| KOG4300 | 1 | 4 | 4 | R | Predicted methyltransferase |
| KOG0749 | 2 | 9 | 4,5 | C | Mitochondrial ADP/ATP carrier proteins |
| KOG0613 | 1 | 5 | 5 | Z | Projectin/twitchin and related proteins |
| KOG0724 | 1 | 5 | 5 | O | Zuotin and related molecular chaperones (DnaJ superfamily), contains DNA-binding domains |
| KOG0806 | 1 | 5 | 5 | E | Carbon-nitrogen hydrolase |
| KOG1637 | 1 | 5 | 5 | J | Threonyl-tRNA synthetase |
| KOG1681 | 1 | 5 | 5 | I | Enoyl-CoA isomerase |
| KOG1700 | 1 | 5 | 5 | TZ | Regulatory protein MLP and related LIM proteins |
| KOG3486 | 1 | 5 | 5 | J | 40S ribosomal protein S21 |
| KOG3591 | 4 | 22 | 5,5 | O | Alpha crystallins |
| KOG0053 | 1 | 6 | 6 | E | Cystathionine beta-lyases/cystathionine gamma-synthases |
| KOG1584 | 1 | 6 | 6 | R | Sulfotransferase |
| KOG3627 | 4 | 25 | 6,25 | E | Trypsin |
| KOG0517 | 1 | 7 | 7 | Z | Beta-spectrin |
| KOG1217 | 1 | 7 | 7 | T | Fibrillins and related proteins containing Ca2+-binding EGF-like domains |
| KOG2392 | 1 | 7 | 7 | V | Serpin |
| KOG3421 | 1 | 7 | 7 | J | 60S ribosomal protein L14 |
| KOG0156 | 2 | 16 | 8 | Q | Cytochrome P450 CYP2 subfamily |
| KOG4295 | 1 | 8 | 8 | O | Serine proteinase inhibitor (KU family) |
| KOG1339 | 1 | 9 | 9 | O | Aspartyl protease |
| KOG3714 | 6 | 61 | 10,16 | O | Meprin A metalloprotease |
| KOG2579 | 2 | 27 | 13,5 | R | Ficolin and related extracellular proteins |

*^a^* KOG ratio is calculated by (number of genes in spider A)/(number of genes in spider B). Spider A is always the one with the bigger number of KOGs in a given category
