# Supplementary material for: Spinning Gland Transcriptomics from Two Main Clades of Spiders (Order: Araneae) - Insights on Their Molecular, Anatomical and Behavioral Evolution
Source: PLoS One. 2011 Jun 29;6(6):e21634. doi: 10.1371/journal.pone.0021634 (PMC3126850; doi:10.1371/journal.pone.0021634)
Supplement: Supporting Information S7 — Repetitive sequences from G. cancriformis spidroins. (DOC) [file pone.0021634.s007.doc]

SUPPLEMENTARY INFORMATION **S7**

Prosdocimi *et al*., 2011. Spinning gland transcriptomics from two main clades of spiders (order: Araneae) - insights on their molecular, anatomical and behavioral evolution.

**Repetitive sequences from *G. cancriformis* spidroins**

Spider silks are known for their highly repetitive sequences; rich in Ala and Gly amino acids. The translation of the unigenes annotated as spidroins allowed the identification of the repetitive parts of many spidroins. Figure S7.1 shows partial repeated sequences for tubulliform and major ampullate spidroin 2 in *G. cancriformis*. The sequences present high sequence similarity to previous published spidroins.


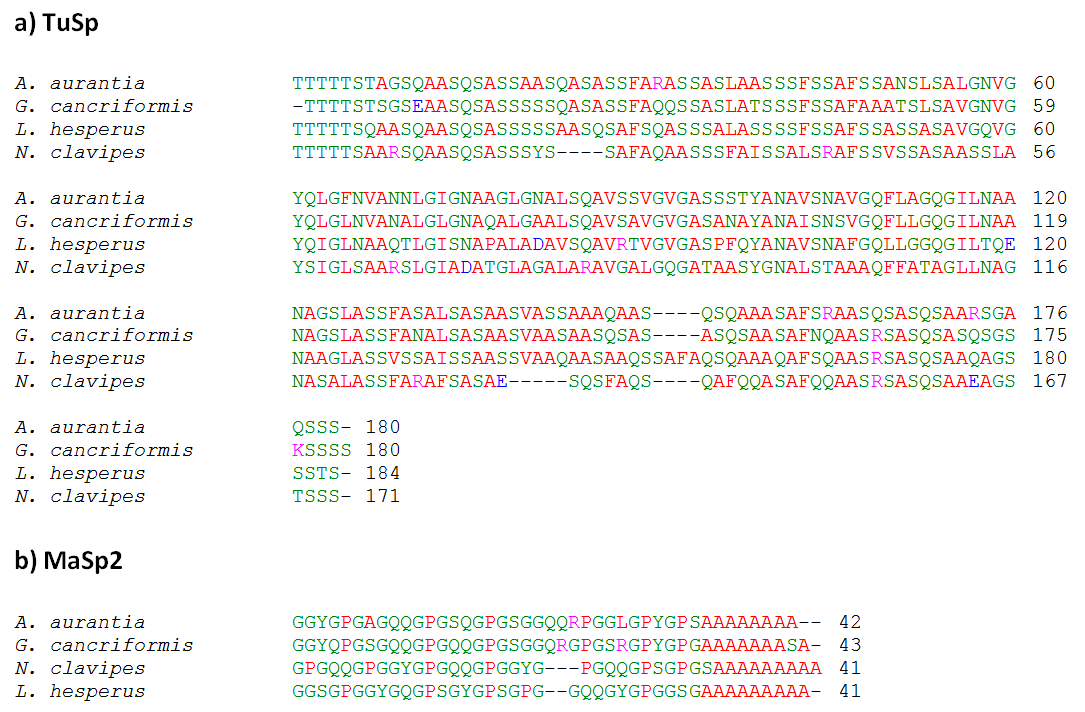


**Figure S7.1**: ClustalW alignments evidencing the repetitive regions found for (a) Tubulliform (TuSp) and (b) Major ampullate spidroin 2 (MaSp2). Data analyzed from the spiders *Argiope aurantia* (AAX45291 and AAK30592), *Nephila clavipes* (AAX45295 and AAT75317), *Latrodectus hesperus* (AAY28931 and ABD66603), and *Gasteracantha cancriformis*.
